# Expression of a Constitutively Active Calcineurin Encoded by an Intron-Retaining mRNA in Follicular Keratinocytes

**DOI:** 10.1371/journal.pone.0017685

**Published:** 2011-03-14

**Authors:** Atsushi Fujimura, Hiroyuki Michiue, Tei-ichi Nishiki, Iori Ohmori, Fan-Yan Wei, Hideki Matsui, Kazuhito Tomizawa

**Affiliations:** 1 Department of Physiology, Okayama University Graduate School of Medicine, Dentistry and Pharmaceutical Sciences, Okayama, Japan; 2 Department of Molecular Physiology, Faculty of Life Sciences, Kumamoto University, Kumamoto, Japan; 3 PREST, Japan Science Technology Agency (JST), Kawaguchi, Japan; University of North Dakota, United States of America

## Abstract

Hair growth is a highly regulated cyclical process. Immunosuppressive immunophilin ligands such as cyclosporin A (CsA) and FK506 are known as potent hair growth modulatory agents in rodents and humans that induce active hair growth and inhibit hair follicle regression. The immunosuppressive effectiveness of these drugs has been generally attributed to inhibition of T cell activation through well-characterized pathways. Specifically, CsA and FK506 bind to intracellular proteins, principally cyclophilin A and FKBP12, respectively, and thereby inhibit the phosphatase calcineurin (Cn). The calcineurin (Cn)/NFAT pathway has an important, but poorly understood, role in the regulation of hair follicle development. Here we show that a novel-splicing variant of calcineurin Aß CnAß-FK, which is encoded by an intron-retaining mRNA and is deficient in the autoinhibitory domain, is predominantly expressed in mature follicular keratinocytes but not in the proliferating keratinocytes of rodents. CnAß-FK was weakly sensitive to Ca^2+^ and dephosphorylated NFATc2 under low Ca^2+^ levels in keratinocytes. Inhibition of Cn/NFAT induced hair growth in nude mice. Cyclin G2 was identified as a novel target of the Cn/NFATc2 pathway and its expression in follicular keratinocytes was reduced by inhibition of Cn/NFAT. Overexpression of cyclin G2 arrested the cell cycle in follicular keratinocytes *in vitro* and the Cn inhibitor, cyclosporin A, inhibited nuclear localization of NFATc2, resulting in decreased cyclin G2 expression in follicular keratinocytes of rats *in vivo*. We therefore suggest that the calcineurin/NFAT pathway has a unique regulatory role in hair follicle development.

## Introduction

The hair follicle is a highly developed organ and the mechanisms that regulate the hair cycle have been extensively investigated [1], [2]. Immunosuppressant drugs such as cyclosporin A (CsA) and FK506, which are indispensable for transplantation, cause hypertrichosis [1], [3]; recent studies have shown that this phenomenon is associated with inhibition of the calcineurin (Cn)/nuclear factor of activated T cells (NFAT) signaling pathway in follicular keratinocytes and follicular stem cells [4], [5].

Cn, a Ca^2+^/calmodulin-dependent serine/threonine phosphatase, plays important roles in cooperation with NFAT in various tissues such as the immune system, cardiac muscle and neurons [6]–[8]. After activation via a rise in intracellular Ca^2+^ concentration, Cn dephosphorylates cytoplasmic NFAT (NFATc1-c4); this results in translocation of NFAT into the nucleus where it binds to DNA to activate gene transcription [9]. In follicular keratinocytes, NFATc2 regulates the expression of cell cycle-associated proteins, including p21^waf1/cip1^ and p27^kip1^, and apoptosis-associated proteins such as p53, and, consequently, controls the transition from the anagen to catagen stages [4]. Similarly, in bulge stem cells, NFATc1 regulates proliferation of follicular stem cells through Cdk4 repression [5].

Cn, which is ubiquitously expressed, is a heterodimer comprised of a catalytic subunit (CnA) binding with calmodulin (CaM) and a calcium-binding regulatory B subunit (CnB) [10]. CnA has four principal domains involved in enzymatic regulation: the N-terminus catalytic domain, the CnB-binding domain, the CaM-binding domain, and the C-terminus autoinhibitory domain. The latter conformationally blocks phosphatase activity and is released from the catalytic domain in response to Ca^2+^/CaM binding [10]. Three isoforms (alpha, beta, gamma) of the CnA subunit have been identified. The alpha and beta (ß) isoforms are expressed in a range of tissues whereas the gamma isoform is predominantly expressed in the testis [11]–[13]. Although eleven splicing variants of CnAß have been identified, only four of these variants appear to have functional phosphatase activity as they contain a conserved catalytic domain (ID No. GC10M074866 in GeneCards). Increased levels of intracellular Ca^2+^ are believed to trigger Cn activation and to be a key regulator of a variety of physiological and pathological processes [10], [14], [15]. In general, follicular keratinocytes are cultured under low Ca^2+^ (<50 µM) conditions and an increase in intracellular/extracellular Ca^2+^ concentration readily induces differentiation [16], [17]. Currently, little is known about Cn activation under such low Ca^2+^ conditions in follicular keratinocytes or of the mechanism of Cn-dependent regulation of the growth of hair follicles. Interestingly, it has been pointed out that Cn is functionally active and activates NFATc2-dependent gene transcription in follicular keratinocytes, resulting in inhibition of the hair cycle [4]. Overall, the molecular mechanism of Cn activation in follicular keratinocytes seems to differ from that in other tissues such as neurons and T cells. In the present study, we show that a novel splicing variant of CnAß(CnAßin follicular keratinocytes, CnAß-FK) is predominantly expressed in follicular keratinocytes. CnAß-FK was deficient in the autoinhibitory domain and was therefore active independently of Ca^2+^ levels. To clarify the roles of CnAß-FK in the hair cycle, we sought to identify the cell cycle regulators whose expression was regulated by CnAß-FK/NFATc2. We found that cyclin G2, an atypical cyclin that correlates with cell cycle arrest, was regulated by CnAß-FK/NFATc2 [18], [19]. Our findings provide the first indication of the role of functionally active Cn in follicular keratinocytes.

## Materials and Methods

### Cell Culture

PHK16-0b (PHK) cells established from human keratinocytes, HEK293 cells and HeLa cells were provided by the Health Science Research Resources Bank (Osaka, Japan). PHK cells were cultured in the serum-free medium EpiLife in the presence of 60 µM CaCl_2_ (Cascade Biologics), supplemented with EDGS (growth supplement, S-012-5, Cascade Biologics), penicillin, streptomycin, and amphotericin B (Nacalai tesque, Japan). At 80% confluence, cells were trypsinized (Trypsin/EDTA, Toyobo) and subcultured onto culture plates coated with human Type-1 collagen (Coating Matrix Kit, Cascade Biologics). HEK 293 cells and HeLa cells were cultured in D-MEM (Gibco) with fetal bovine serum (10%) and antibiotics.

### Cloning of a Splicing Variant of CnAßCnAß-FK)

A cDNA library was constructed from PHK cells by Takara Bio (Ohtsu, Japan). Briefly, cDNAs were synthesized from PHK cells using an Oligo(dT)-Anchor primer (Takara Bio) and subcloned into the EcoRI/XhoI sites of pBluescript II SK(+) plasmids. The full nucleotide sequence of CnAß-FK was determined by PCR and nested-PCR using the primers listed in Tables S1-A and -B. PCR products were cloned into pCR-Blunt II-TOPO (Invitrogen) and analyzed using the listed sequencing primers (Table S1-a and -b). For further determination of the C-terminus, 3′-RACE (Rapid Amplification of cDNA Ends) was performed with the 3′ Full RACE Core Set (Takara Bio) (Table S1-c). mRNAs were isolated from PHK cells using the FastTrack 2.0 kit (Invitrogen) according to manufacturer's protocol. Details of the primer sequences and cloning process are given in Table S1. PCR was performed using the KOD FX DNA polymerase (Toyobo).

### Calcineurin Phosphatase Assay

Calcineurin activity was determined by a calcineurin phosphatase assay that measures dephosphorylation of p-nitrophenol phosphate (pNPP). HEK293 cells were transfected with pcDNA3.1 plasmid (Invitrogen) containing human CnAß-FK cDNA. After 24 h, the cells were harvested and lysed in a buffer comprised of 20 mM Tris-HCl (pH 7.2), 1 mM EGTA, 100 mM sodium chloride, 1% Triton X-100, and Protease Inhibitor Cocktail (Roche). Mouse brain and PHK cells were lysed in the same buffer. After centrifugation at 65,000 *g*, each supernatant (1 mg of total protein) was immunoprecipitated using 10 µg of polyclonal anti-CnAß antibodies (Chemicon). Immunoprecipitated samples were incubated on 96-well microplates in a Cn assay buffer (50 mM Tris-HCl (pH 7.2), 1 mg/ml CaM, 1% BSA. 1 µM okadaic acid) in the presence or absence of 2 mM CaCl_2_, 2 mM EGTA or 1 µM CsA at 37°C for 15 min. After adding 1 mg/ml pNPP, the samples were further incubated for 15 min. The reaction was stopped by adding sodium hydroxide (final concentration, 300 mM), then the absorbance at 405 nm was measured with a microplate reader. Purified bovine CaN was purchased from Upstate (14-390). In each sample, the data were standardized against Ca^2+^ stimulation data.

### Animal Studies

The effect of 11R-VIVIT on hair growth *in vivo* was investigated in male nude mice aged 28 days (BALB/c Slc-nu, Shimizu Laboratory Supplies, Kyoto). For drug application, 11R-VIVIT (10 mg) was dissolved in 1 g hydrophilic ointment (Merck). Twenty-five mg of the ointment containing 250 µg of 11R-VIVIT was applied to the skins of the mice once per day. After application of the ointment for seven days, the animals were killed, and the skins excised and immediately fixed with 4% paraformaldehyde (PFA) at 4°C overnight. As a control, mice were treated as above, but with ointment lacking 11R-VIVIT.

To investigate the effect of CsA, Wistar rats aged 24 days (Shimizu Laboratory Supplies) were intraperitoneally injected with 100 mg/kg CsA on 3 successive days. The animals were then killed and the skins then excised and used for immunohistochemistry. As a control, rats were injected with vehicle only. All procedures were approved by the Ethics Review Committee for Animal Experimentation of our institute (OKU-2009192).

### *In Situ* Hybridization

In situ hybridization was carried out as described previously [20]. Briefly, probes for detecting rat CnAß-FK mRNA were prepared by first cloning the full sequence of intron 11 of CnAß-FK cDNA (Figure 2A) into pCR-Blunt II-TOPO (Invitrogen). The plasmid was linearized and transcribed with SP6 or T7 RNA polymerase. Digoxigenin-UTP-labeled RNA probes were generated from the DNA template using a DIG RNA labeling kit (Roche). Fresh frozen sections (6 µm thickness) were prepared on silane-coated glass microscope slides and immediately fixed with 4% paraformaldehyde (PFA) and 0.1% glutaraldehyde (GA) in 0.1 M phosphate buffer (PB, pH 7.4) for 15 min. Prior to hybridization, RNA probes were denatured at 80°C for 5 min. Hybridization was performed at 60°C for 16 h with a digoxigenin-labeled probe (1 µg/ml) in hybridization buffer (50% formamide, 2× SSC, 1% SDS). After a series of wash steps, the single-stranded RNA probes were removed with RNase A (10 µg/ml) at 37°C for 30 min. The sections were incubated with alkaline phosphatase-conjugated anti-DIG antibody and hybridization signals were detected by an NBT/BCIP colorimetric reaction according to manufacturer's recommendation (Roche). For specificity control, a sense probe was used.

### Immunofluorescent Staining

Skin sections from male Wistar rats were immunofluorescently stained as follows. Excised skin samples were immediately frozen in Optimal Cutting Temperature compound (Sakura Finetek, Japan) and sequentially sectioned at a thickness of 10 µm. The sections were fixed with 4% PFA in 0.1 M phosphate buffer (pH 7.4) for 15 min and then incubated with 5% normal goat serum (Abcam) in blocking buffer (1× PBS, 0.3% Triton X-100) at room temperature for 1 h. After washing in PBS, the sections were incubated with primary antibodies in dilution buffer (1× PBS, 10% BSA, 0.3% Triton X-100) at 4°C overnight. The sections were incubated with secondary antibodies in dilution buffer at room temperature for 2 h in a dark box. The sections were then washed, stained with Hoechst 33258 (1 µg/ml) for 2 min, and viewed using a confocal microscope (FluoView™ FV300, OLYMPUS). The following primary and secondary antibodies were used: NFATc2 (Upstate, 07-136, rabbit antiserum, 1∶100), NFATc1 (Santa Cruz, 7A6, mouse, 1∶100), and cyclin G2 (Abcam, ab5502, rabbit polyclonal, 1∶200). Anti-mouse or anti-rabbit IgG (Molecular Probes, Alexa Fluor 488- or 555- conjugated goat IgG, 1∶200) were used as secondary antibodies.

For immunocytochemistry, PHK cells were cultured on glass-bottomed dishes coated with human Type-1 collagen and grown to 80% confluence. After incubation in high [Ca^2+^] medium for 4 h, the cells were immediately fixed with 4% PFA in PB, permeabilized with 0.5% Triton X-100 and blocked with 10% BSA. The following primary and fluorochrome-conjugated secondary antibodies were used: NFATc2 (Upstate, 07-136, rabbit antiserum, 1∶250) and anti-rabbit IgG (Molecular Probes, Alexa Fluor 488-conjugated goat IgG, 1∶500).

### Western Blotting Analysis

Skins were excised from male Wistar rats at postnatal day 7 and the epidermal and dermal layers were separated with dispase at 4°C overnight. The isolated samples were frozen in liquid nitrogen, pulverized and lysed in lysis buffer, followed by boiling in sample buffer (50 mM Tris-HCl, pH 6.8, 5% glycerol, 1% SDS, 0.1% BPB, 2% 2-ME). PHK 16-0b cells and HEK293 cells were harvested at 80% confluence. After sonication, cell lysates were boiled in sample buffer. The cell and tissue lysates were separated by SDS-PAGE on a 10% acrylamide gel and transferred to a nitrocellulose membrane using the iBlot Dry Blotting System (Invitrogen). After blocking with 5% skim milk in TBST, the blots were incubated with primary antibodies against the following proteins: CnA (epitope: the middle (246–265) of the amino acid sequence, SPA-610, Stressgen Bioreagents Corp.), CnAß (epitope: the N-terminus (18–28) of the amino acid sequence, AB1697, Lot No. 0612047223, Chemicon), cyclin G2 (1572-1, Epitomics and ab5502, Abcam), p21 (sc-756, Santa Cruz), ß-actin (A-5316, Sigma), and GAPDH (sc-32233, Santa Cruz). These primary antibodies and HRP-conjugated secondary antibodies (Sigma) were appropriately diluted according to manufacturer's recommendation.

### Microarray Analysis

Microarray analysis was performed using a human 44K whole genome oligo DNA chip (Agilent Technologies) as described previously [21]. Briefly, mRNAs (150 ng) from PHK cells treated with 1 µM CsA or 3 µM 11R-VIVIT for 24 h were pooled into one master total RNA mix, and labeled with Cy-3 or Cy-5 using an Agilent Low RNA Input Fluorescent Linear Amplification Kit (Agilent). Hybridization and wash processes were performed according to the manufacturer's instructions, and hybridized microarrays were scanned using an Agilent Microarray scanner G2565BA. For detection of genes with significant differential expression between the control group and those treated with CsA or 11R-VIVIT, each slide image was processed using the Agilent Feature Extraction ver.8.5.1.1. All data is MIAME compliant and the raw data has been deposited in a MIAME compliant database, Gene Expression Omnibus (accession number GSE25175).

### Proliferation Assay and Vector Constructs

The effect of cyclin G2 expression on keratinocyte proliferation was analyzed in PHK cells using a WST-1 assay (Roche) as described previously [22]. Recombinant adenovirus containing human *ccng2* (cyclin G2) cDNA, chicken ß-actin promoter and SV40 polyadenylation signal was produced using the Adenovirus Expression Vector Kit (Takara) as described previously [22]. Human *ccng2* gene was purchased from ATCC (item number, 9117362; GenBank, BC032518). The LacZ recombinant adenovirus was also produced following the manufacturer's protocol. For the transient gene expression of CnAß-FK in HeLa cells, the open reading frame of CnAß-FK cDNA was subcloned into the pcDNA 3.1/V5-His vector (Invitrogen) using a TOPO TA Expression Kit (Invitrogen) and the cells were transfected with the plasmids using Lipofectamine 2000 (Invitrogen).

### Statistics

Data are shown as means ± S.E. The data were analyzed either by Student's *t* test to identify significant differences between two groups or by Scheffe's posttest analysis following two-way ANOVA to compare multiple groups. P values less than 0.05 were considered significant.

## Results

### Molecular Characteristics of a Novel Calcineurin Aß Isoform

**Figure 1:**
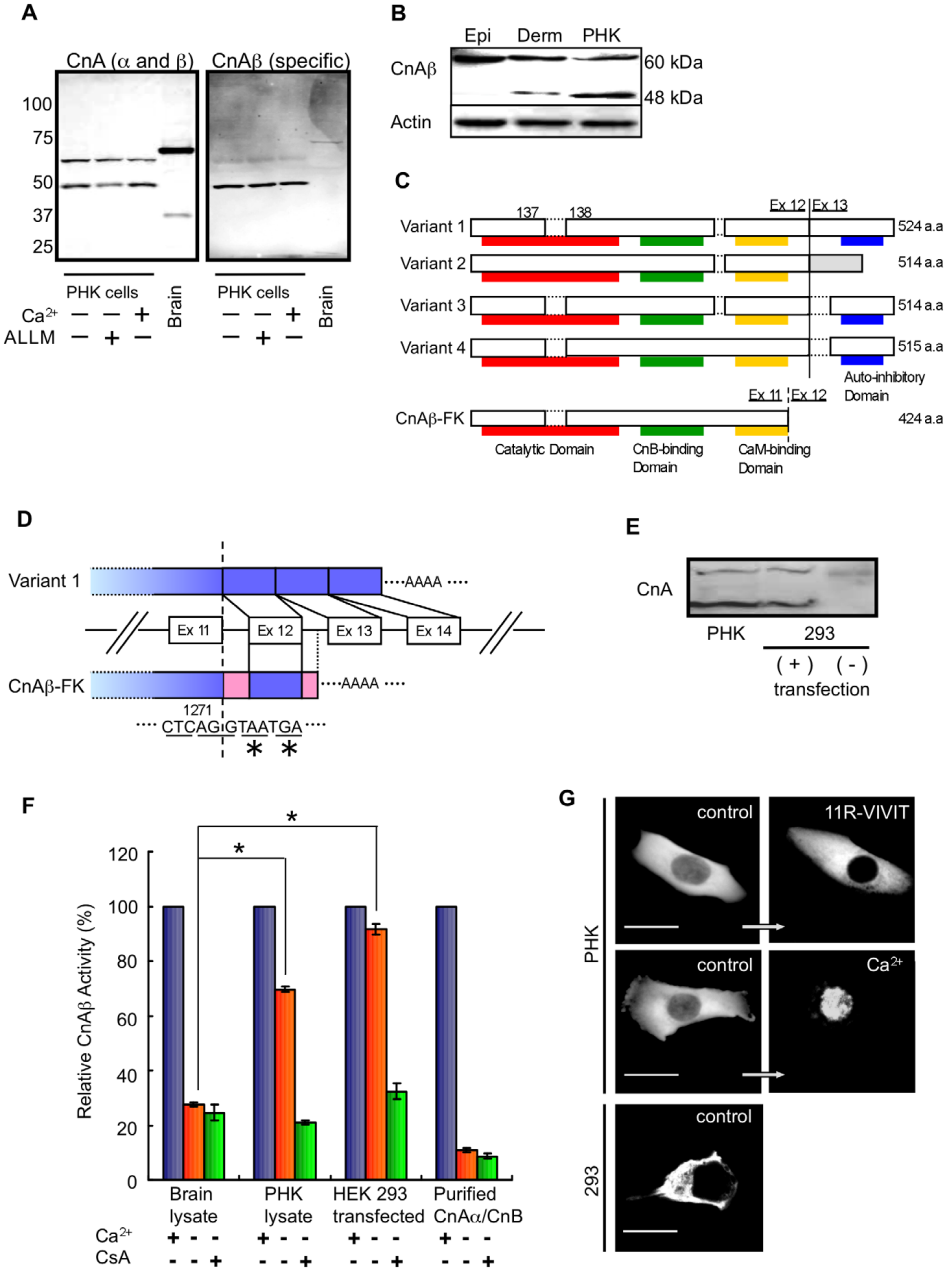
Molecular characteristics of a novel variant of CnAß (CnAß-FK). (A) CnA expression in PHK cells and mouse brain. PHK cells were treated with 1 µM ALLM or incubated with 1 mM Ca^2+^ for 24 h. (B) Expression of CnAß in the dermis and epidermis of rat. An atypical CnAß with a molecular mass of 48 kDa was observed in the dermis and PHK cells. ß -actin expression (Actin) was used as the control. (C) Comparison of the primary structure of CnAß-FK with other splicing variants of CnAß. CnAß-FK lacks an autoinhibitory domain. The gray box in variant 2 represents amino acid sequences translated from mRNAs of the intron. (D) Comparison of the nucleotide sequence of the 3′ terminus of CnAß-FK cDNA with that of variant 1. Blue box, cDNA derived from exons; red box, cDNA derived from introns. *, stop codon. (E) Ectopic expression of CnAß-FK in HEK293 cells. PHK cells lysate was loaded as a positive control. (F) Relative CnAß activity in brain lysate and each cell line. HEK 293 cells were transfected with pcDNA3.1 plasmid containing CnAß-FK cDNA and harvested 24 h later. Cn phosphatase activity was assayed in the presence or absence of 1 mM Ca^2+^ or 1 µM cyclosporin A (CsA). Phosphatase activity of each sample was standardized with that in the presence of Ca^2+^ but not CsA. Purified CnA from bovine brain was used as a positive control. n = 5 each sample. *P<0.01. (G) Subcellular distribution of NFATc2 in PHK cells and HEK293 cells. Cells were transfected with a GFP-NFATc2 plasmid and incubated for 24 h. PHK cells were then treated with 11R-VIVIT (1 mM) for 6 h or stimulated with Ca^2+^ (1 mM) for 15 min. Note that GFP signals were seen not only in the cytoplasm but also in the nucleus of control PHK cells. In HEK293 cells, under basal conditions, GFP signals were seen only in the cytoplasm. Scale bar, 10 µm.

To investigate the expression of calcineurin A (CnA) in hair follicles (HF), we first examined CnA expression in primary human keratinocyte (PHK) cells. Western blot analyses using anti-CnA antibodies, which recognize the alpha and ß isoforms of CnA, identified two bands in PHK cells: an atypical CnA band (∼48 kDa) and the typical CnA (∼60 kDa) band (Fig. 1A). The atypical CnA band was not observed in other organs including mouse brain (Fig. 1A), liver, heart, kidney and lung (data not shown). This band could stably be detected using anti-CnAß antibodies (Fig. 1A), suggesting that it represents a ß isoform of CnA. We previously reported that calpain, a Ca^2+^-activated neutral cysteine protease, cleaved CnA alpha and generated a truncation product with a molecular mass of ∼48 kDa in excessively excited neurons and in Alzheimer's brain tissue [15], [23]. We therefore postulated that the atypical CnAß was a truncated product of CnAß produced by calpain. To investigate this possibility, PHK cells were treated with ALLM, a calpain inhibitor. However, the expression of the 48 kDa and 60 kDa CnAβ were not changed by this treatment (Fig. 1A). Moreover, Ca^2+^ stimulation did not alter 48 kDa CnA expression and no additional bands were observed in PHK cells (Fig. 1A). These results suggested that the atypical CnA was not generated by calpain. Next, we determined whether the atypical CnAß was expressed in dermis containing HF and in epidermis without HF in rat skin. Both the atypical and typical CnAß were observed in rat dermis whereas only typical CnAß was expressed in rat epidermis (Fig. 1B). These results suggested that the atypical CnAß might be expressed in hair follicles but not in the epidermis.

To investigate the molecular characteristics of the atypical CnAß, we constructed a cDNA library from PHK cells and cloned a splicing variant of CnAß (CnAß-FK) corresponding to the 48 kDa CnAß. Sequence analysis of the CnAß-FK cDNA revealed that it was composed of CnAß coding sequence from exons 1–11 (1271 bp), intron 11 (1669 bp), exon 12 (98 bp), and part of intron 12 (930 bp) ending with a poly A tail (Fig. S1). A stop codon was present at the junction of exon 11 and intron 11 (Figure 1D & S1). As a result, the intron-retaining mRNA encoded a truncated CnAß that lacked the autoinhibitory domain (Figure 1C & S2).

It was previously shown that artificial truncated form of CnA, which also lacked an autoinhibitory domain, had Ca^2+^-independent Cn activity [15], [24]. To investigate whether CnAß-FK showed Ca^2+^-independent activity, we examined Cn activity in PHK and 293 cells overexpressing CnAß-FK (Fig. 1E & 1F). CnAß-FK was stably expressed in the cells (Fig. 1E). Cn activity of a brain lysate and of purified Cn was Ca^2+^-dependent, whereas PHK cells had high Cn activity under low Ca^2+^ conditions (Fig. 1F). In cells overexpressing CnAß-FK, moreover, the activity was weakly sensitive to Ca^2+^ (Fig. 1F). CnAß-FK activity was inhibited by cyclosporin A (CsA), a potent inhibitor of Cn.

The subcellular distribution of NFAT was examined by optical imaging in PHK and HEK293 cells that had been transfected with GFP-NFATc2 cDNA. In HEK293 cells lacking CnAß-FK, NFAT was located in the cytoplasm (Fig. 1G). In intact PHK cells, on the other hand, NFAT was observed in both the nucleus and cytoplasm (Fig. 1G). In PHK cells treated with the membrane-permeable specific NFAT inhibitor, 11R-VIVIT, which inhibits nuclear translocation of NFAT by blocking the NFAT-Cn interaction [25], [26], NFAT was observed in the cytoplasm but not in the nucleus (Fig. 1G). In contrast, PHK cells stimulated with Ca^2+^ showed translocation of NFAT to the nucleus (Fig. 1G). Moreover, the pattern of distribution of endogenous NFATc2 was observed in PHK cells under a low Ca^2+^ (40 µM) concentration. Endogenous NFATc2 was observed in both the nucleus and cytoplasm (Fig. S3). Our observations suggest that Cn has high activity in PHK cells regardless of intracellular Ca^2+^ levels and induces NFAT translocation into the nucleus.

### Localization of CnAß–FK mRNA in Rat Skin

**Figure 2:**
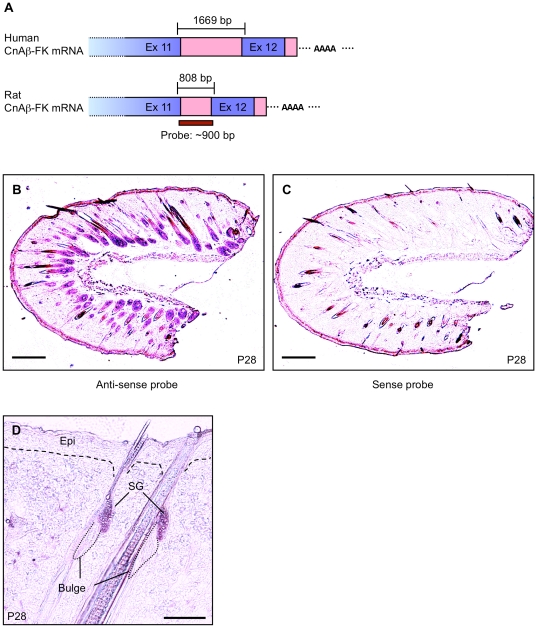
Localization of CnAß -FK mRNA in rat skin. (A) Schema of sense and anti-sense probes used for *in situ* hybridization (ISH). The probes correspond to the full sequence of intron 11 and are specific for CnAß-FK mRNA. Sense probes were used as the negative control. (B and C) Expression of CnAß-FK mRNA in rat skin. Skin sections from the parietal region were prepared from male Wistar rat (postnatal day 28, corresponding to the late phase of the second anagen). (D) Higher magnification micrograph to show the mRNA expression in epidermais and SG. Epi, epidermis; SG, sebaceous gland. Scale bars in B and C, 1 mm; in D, 50 µm.

**Figure 3:**
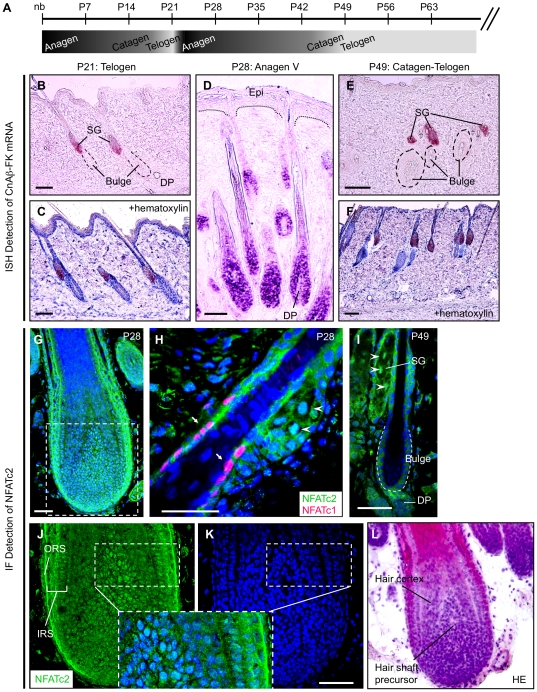
CnAß-FK mRNA and NFATc2 Localizations in rat skin. (A) Time-scale of the hair cycle in male Wister rats. Each phase was determined using criteria described previously [42]. The intensity of the gray shading indicates the rate of proliferation of follicular keratinocytes: white<proliferation activity<black. (B–F) Distribution of CnAß-FK mRNA. B and C, telogen stage. D, late phase of anagen stage. E and F, transition stage from catagen to telogen. (C and F) Sections were counter-stained using hematoxylin (blue staining). (G–J) Results of immunohistochemical analysis of NFATc2 (green) and NFATc1 (red) in hair follicles. Cell nuclei were visualized by Hoechst staining (blue in G–I and K). (G) NFATc2 expression in hair follicle at postnatal day 28. (H) NFATc2 and NFATc1 expression in bulge and SG cells at postnatal day 28. Arrows, NFATc1 expression in bulge cells. Arrowheads, nuclear localization of NFATc2 in SG cells. (I) NFATc2 expression at postnatal day 49. Arrowheads indicate localization of NFATc2 in nuclei of SG cells. (J and K) Higher magnification image of the boxed region in G. (J) NFATc2 expression. (K) Hoechst staining. (L) Hematoxylin-eosin (H.E.) staining of hair follicles at postnatal day 28. After immunofluorescence staining, H.E. staining was performed in the same section to reveal the histology of hair follicle. DP, dermal papillae; IRS, inner root sheath; ORS, outer root sheath; SG, sebaceous gland. Scale bars in B–F, 100 µm; in B, C, and G–K, 50 µm.

Immunohistochemical analysis using CnAß antibodies showed that CnAß was strongly expressed in both epidermal and follicular keratinocytes of the rat skin (Fig. S4). CnAßwas also observed in the cells of the outer root sheath (ORS) and the inner root sheath (IRS) but was undetectable in other cells (Fig. S4). However, the immunohistochemical analysis does not distinguish signals from typical CnAß (∼60 kDa) and CnAß-FK. Therefore, we performed an *in situ* hybridization (ISH) using a specific anti-sense probe to CnAß-FK mRNA to verify the distribution of CnAß-FK mRNA in rat skin (Fig. 2A). During the late phase of the anagen stage (P28), in which almost follicular keratinocytes are mature, CnAß-FK mRNA was strongly expressed in follicular keratinocytes of the hair shaft and matrix (Fig. 2B). It was also strongly expressed in IRS cells but was not expressed in ORS cells or the dermal papillae of hair follicles (Fig. 2B and 3D). Strong mRNA expression was also seen in cells of the sebaceous gland (SG) (Fig. 2D). In contrast, no CnAß-FK mRNA was detected in either bulge stem cells or epidermal keratinocytes (Fig. 2D). Interestingly, at an early phase of the anagen stage (P25) and at the transition from telogen to anagen stages (P24), when immature follicular keratinocytes in hair germ (HG) are proliferating, CnAß-FK mRNA was not expressed in the keratinocytes of the HG (Fig. S5A–D). At these stages, high levels of mRNA expression were only detected in the SG (Fig. S5A–D). Moreover, at the catagen and telogen stages, when follicular keratinocytes were absent, CnAß-FK mRNA expression was restricted to SG cells (Fig. 3B, C, E and F). CnAß-FK was also expressed in human hair follicle (Fig. S6). These results agree with those from the western blotting analysis shown in Fig. 1B, and suggest that CnAß-FK may be predominantly expressed in mature follicular keratinocytes and SG cells.

If CnAß-FK is constitutively active as shown in Fig. 1, then it is possible that some NFATc2 might be localized in the nuclei of mature follicular keratinocytes and SG cells. To investigate this hypothesis, we determined the subcellular distribution of NFATc2 at each hair cycle stage in rat skin. At the transition from the catagen to telogen stages (P49), NFATc2 was present in the nuclei of cells of the SG (arrowheads in Fig. 3I), whereas it was expressed in the cytoplasm but not the nuclei of dermal papilla cells and follicular keratinocytes (Fig. 3I). Nuclear localization of NFATc2 was also observed in SG cells during the late phase of the anagen stage (arrowheads in Fig. 3H). Moreover, NFATc2 was strongly expressed in the nuclei of mature follicular keratinocytes at the late phase of the anagen stage (Fig. 3G, J–L). NFATc1 was specifically expressed in bulge stem cells (Fig. 3H) as previously reported [5]. At P24 and P25, NFATc2 was observed in the cytoplasm of proliferating follicular keratinocytes in which CnAß-FK was not expressed (compare Fig. S5A and C with Fig. S5E and F). These results suggest that CnAß-FK localized in mature keratinocytes and SG cells, and that a consequence of this localization was the translocation of NFATc2 in the cell nuclei.

### Effect of NFAT Inhibition on Hair Growth in Nude Mice

**Figure 4:**
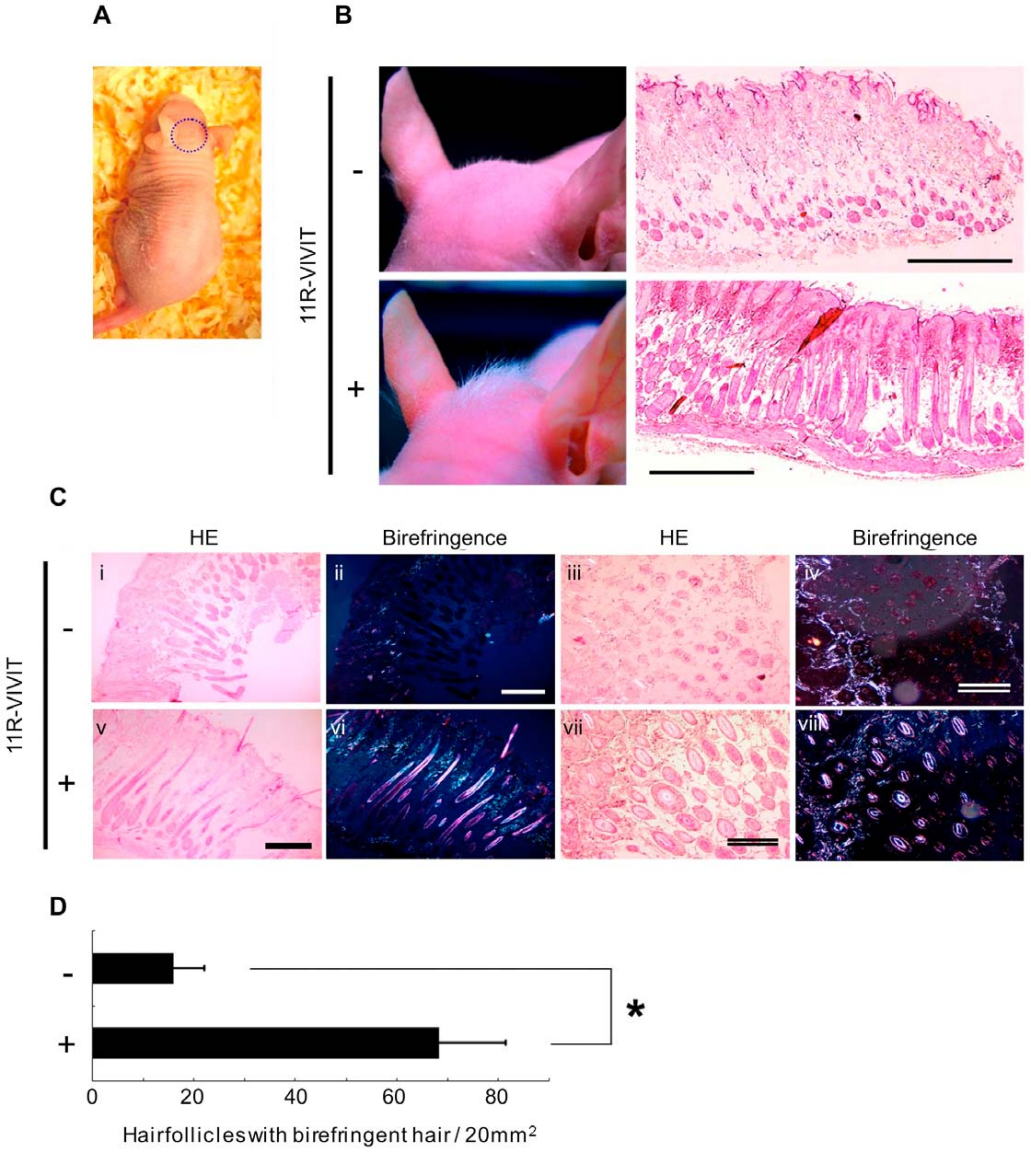
Effects of 11R-VIVIT on hair growth induction in nude mice. (A) The vertex region (blue circle) was chosen as the treatment spot with 11R-VIVIT. (B) Macroscopic and microscopic assessment of hair growth induction. Upper panels, control mouse treated with ointment only. Lower panels, 11R-VIVIT-treated mouse. Right panels, longitudinal sections stained with H. E. Scale bar, 1 mm. (C) Histological features of the skin of control and 11R-VIVIT-treated mice. i–iv, control mice; v–viii, 11R-VIVIT-treated mice. Longitudinal (i, ii, v and vi) and transverse (iii, iv, vii and viii) sections stained with H.E. (i, iii, v and vii), and the same sections assessed for birefringent hair shafts using a polarizing microscope (ii, iv, vi and viii). Scale bars in ii and v, 500 µm; in iv and vii, 200 µm. (D) Hair growth in control (−) and 11R-VIVIT-treated (+) mice was quantified using a polarizing microscope by counting the number of hair follicles containing birefringent hair shafts. n = 10 each. *P<0.0005.

Cyclosporin A (CsA) and FK506 are widely used immunosuppressants; both have the well-known side effect of causing hypertrichosis [1]. The Cn/NFAT pathway is involved in the mechanism of this side effect [1], [3]. We gave a topical application of an ointment containing 11R-VIVIT to the vertex of nude mice on seven successive days from postnatal day 28 (late phase of the anagen stage). Significant hair growth was observed in the area treated with ointment containing 11R-VIVIT (Figure 4B). Histological analysis of skin specimens from control nude mice revealed dystrophic follicles containing fragmented hair shafts that did not emerge from the hair follicles, and showed little birefringence of the cuticle (Fig. 4C). In contrast, treatment with 11R-VIVIT resulted in relatively normal follicles containing well-differentiated straight hair shafts that emerged from hair follicles and reached the surface of the skin, and showed clear birefringence of the cuticle (Fig. 4C). Moreover, the number of hair follicles containing birefringent hair was significantly increased in 11R-VIVIT-treated mice compared to the control mice (Fig. 4D). These results suggest that inhibition of CN/NFAT signaling pathway enhanced hair growth.

### Involvement of Cyclin G2 in Cell Cycle Arrest in Follicular Keratinocytes

**Figure 5:**
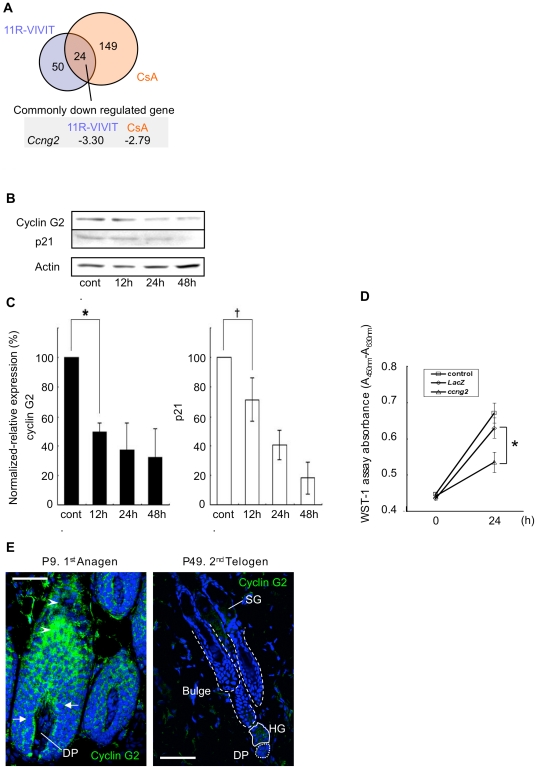
Cyclin G2 is a target molecule of Cn/NFATc2 in follicular keratinocytes. (A) The microarray analysis identified 24 genes that showed down-regulated expression in PHK cells treated with either 11R-VIVIT or CsA; *ccng2* (cyclin G2) expression showed the greatest decrease after Cn/NFAT inhibition. (B) Time-dependent changes in cyclin G2 and p21^waf/cip1^ expression in PHK cells treated with 11R-VIVIT. (C) Quantitative analysis of the changes in expression changes of cyclin G2 and p21^waf/cip1^ in PHK cells after 11R-VIVIT treatment. n = 5 for each sample group. * P<0.01, ^†^ P<0.05. (D) Effect of cyclin G2 overexpression on the proliferation of PHK cells. Cells were infected with recombinant adenoviruses carrying cyclin G2 and lacZ at an MOI of 100. Overexpression of cyclin G2 significantly inhibited cell proliferation compared with *LacZ*-infected cells (*P<0.01). (E) Expression of cyclin G2 in rat hair follicles at postnatal days 9 (first anagen stage) and 49 (second telogen stage). Arrowheads, differentiated keratinocytes in a distal part of the hair shaft; arrows, proliferating keratinocytes in the proximal hair shaft. DP, dermal papillae; HG, hair germ; SG, sebaceous gland. Scale Bar = 50 µm.

As shown in Figure 1G, NFATc2 is a target of CnAß-FK. NFATc2 has been shown to regulate the hair cycle by determining the level of expression of cell cycle-associated proteins and apoptosis-associated proteins [4]. To investigate the mechanism of regulation of the hair cycle after activation of CnAß-FK/NFATc2, we carried out a DNA microarray analysis to identify genes down-regulated by 11R-VIVIT and CsA in PHK cells. Twenty-four genes showed repressed expression after 11R-VIVIT and CsA treatments (Fig. 5A). Of these genes, the largest effect was shown by *ccng2* (Fig. 5A), which encodes the unconventional cyclin G2. Cyclin G2 is an atypical cyclin that associates with protein phosphatase 2A but not proteins of the cell cycle-associated Cdk family; it is involved in cell cycle arrest and apoptosis [18], [19], [27]. The effect of 11R-VIVIT on time-dependent changes in cyclin G2 expression was examined in PHK cells. Cyclin G2 expression decreased with time following 11R-VIVIT application in a similar fashion to that of p21^waf/cip1^, which has been identified as a target protein of Cn/NFAT in the hair follicle (Fig. 5B and C). We further examined whether overexpression of CnAß-FK induced endogenous cyclin G2 in Hela cells. Exogenous CnAß-FK induced the cyclin G2 expression (Fig. S7). Moreover, overexpression of cyclin G2 inhibited the proliferation of PHK cells compared to control and *LacZ*-overexpressing cells (Figure 5D).

Cyclin G2 was strongly expressed in the mature keratinocytes localized distally in the hair shaft (arrowheads), whereas the protein was not observed in proliferating keratinocytes in the proximal part of the hair shaft (arrows) at the first anagen stage (Fig. 5E). In HF at the telogen stage, cyclin G2 expression was weak in the hair germ region and was undetectable in bulge stem cells and in DP cells. In contrast, cyclin G2 was detected in SG cells where CnAß-FK mRNA was detected at the telogen stage (Fig. 5E). We also screened skin from Wistar rats that had been treated with CsA for three successive days from postnatal day 24 (anagen stage) for the subcellular localization of NFATc2 and cyclin G2 expression (Fig. S8). In vehicle-treated rats, NFATc2 was observed in both nuclei (arrows in Fig. S8C) and cytoplasm of follicular keratinocytes in the HF. Cyclin G2 expression was also detected in many of the follicular keratinocytes in the HF of control rats (Fig. S8E). In contrast, NFATc2 was mainly detected in the cytoplasm of follicular keratinocytes of CsA-treated rats (Fig. S8F and G). A low level of cyclin G2 expression was observed in the follicular keratinocytes of CsA-treated rats (Fig. S8I).

## Discussion

The physiological functions of the CN/NFAT signaling pathway have been investigated in diverse organs including the immune system, the neuronal system and the hair follicle. In the hair follicle, the pathway is functionally active and its inhibition enhances hair growth [4], [5]. A recent study elucidated many details of the molecular mechanism of this pathway in hair stem cells [5]. It was found that NFATc1 was specifically expressed and that the Cn/NFATc1 pathway down-regulated proliferation of hair stem cells [5]. However, the precise molecular mechanisms involved in the signaling pathway in follicular keratinocytes remained unclear. In the present study, we cloned a novel splicing variant of CnAß (CnAß–FK) in follicular keratinocytes. CnAß–FK lacked the autoinhibitory domain, resulting in a constitutively active form, and was localized in the nucleus of mature follicular keratinocytes along with NFATc2. In contrast, CnAß–FK was not expressed in immature keratinocytes of the hair germ. These results suggest that constant activation of the CN/NFAT signaling pathway in mature keratinocytes is involved in the regulation of hair growth. Normal hair growth requires a balance between keratinocyte growth and differentiation in the hair follicle [2]. CnAß–FK may be involved in inhibition of follicular keratinocyte growth and the induction of differentiation of the cells. One of the most common side effects of treatment with the immunosuppressant drug CsA is hypertrichosis; this condition affects 50–80% of transplant recipients treated with the drug [28]. In hair follicle stem cells, inhibition of the Cn/NFATc1 pathway by CsA induced the proliferation of stem cells [5]. Overall, the results of previous studies and those described here suggest that CsA inhibits both the CnAß–FK/NFATc2 pathway in follicular keratinocytes and the Cn/NFATc1 pathway in follicular stem cells, resulting in excessive hair growth.

CnAß-FK is encoded by an intron-retaining mRNA and is predominantly expressed in follicular keratinocytes. Under ordinary intracellular conditions, intron-containing pre-mature mRNAs are confined within the nucleus, or are degraded by the cellular nonsense-mediated decay system when transported to the cytoplasm; both of these mechanisms result in restriction of their translation [29], [30]. However, several recent studies have indicated the existence of a mechanism for export of pre-mature mRNAs from the nucleus [31], [32], and also suggested that these mRNAs may play physiologically important roles in addition to being involved in pathological conditions [33]–[35]. Our results here provide the first evidence that an intron-containing pre-mature mRNA has a physiological function in the regulation of hair follicle development. Although the mechanisms of pre-mRNA nuclear export have been investigated, the details remain unclear [36], [37]. Further study is needed to determine the effects of intron 11 and 12 of CnAß on mRNA stability and on expression of CnAß-FK. In the present study, we also found that CnAß-FK was strongly expressed in cells of the sebaceous gland. There have been no previous reports that Cn might have a function in the sebaceous gland. It would be interesting to investigate the physiological functions of CnAß-FK in the sebaceous gland. Another extension of research on this variant would be to investigate the mechanisms of alternative splicing and posttranscriptional modification.

Central dogma states that Cn activity in mammals is tightly regulated by Ca^2+^ through CnB and calmodulin binding. In response to changes in intracellular Ca^2+^ levels, the autoinhibitory domain of CnA is removed and the catalytic site is exposed [38]. We showed previously that the autoinhibitory domain of CnA is cleaved by calpain and that Cn becomes the constitutively active form during neurodegeneration [15], [23]. This pattern of cleavage is also observed in patients with diseased myocardium [39], suggesting that Cn changes the constitutively active form in some pathological conditions. However, the present study demonstrated that CnAß-FK was a normally occurring variant and was the constitutively active form with a physiological function in mature follicular keratinocytes. Moreover, a splicing variant form of CnAß (variant 2 in Fig. 1C) has been cloned and is also encoded by an intron-retaining mRNA [40]. In variant 2 of CnAß, the autoinhibitory domain has been replaced with a unique C-terminal region generated by the translation of intron 12 (41 amino acids); consequently, variant 2 is also constitutively active [41]. A recent study showed that variant 2 is highly expressed in proliferating myoblasts and regenerates skeletal muscle fibers [41]. Together, these findings indicate that constitutively active variants of Cn may play important roles in the regulation of physiological functions such as cell proliferation and differentiation in various organs and tissues.

The present study showed that CsA blocked the Cn activity of CnAß-FK. This result agrees with that of truncated CnA by calpain [15], [23]. In contrast, variant 2 of CnAß has reduced sensitivity to the action of immmunosuppressants [41]. Variant 2 of CnAß has a unique C-terminal region generated by the translation of intronic sequences. The alternative C-terminal domain in variant 2 of CnAß may interfere with the binding of the immunosuppressant- immunophilin complex to Cn and prevent its inactivation. The side effects of immunosuppressants are seen in hair follicles and SG as hypertrichosis and hyperplasia but not in skeletal muscle. Differences in sensitivity to immunosuppressants between CnAß-FK and variant 2 of CnAß might determine the nature of any side effects of immunosuppressant treatment.

We showed here that cyclin G2 is a novel target of Cn/NFATc2 in follicular keratinocytes. Cyclin G2 is an atypical cyclin involved in cell cycle arrest and apoptosis [19], [27]. Previous studies identified the cyclin-dependent kinase inhibitors p21^waf/cip1^ and p27^kip1^ as targets of Cn/NFATc2 in follicular keratinocytes [4], [16]. Both proteins inhibit proliferation and induce differentiation of keratinocytes [4]. Overall, these observations suggest that activation of the Cn/NFATc2 signaling pathway is involved in the induction of the expression of some signaling molecules that inhibit proliferation and induce terminal differentiation in keratinocytes. Therefore, immunosuppressants may induce proliferation of follicular keratinocytes and block terminal differentiation in these cells.

In conclusion, the data we present here demonstrate a novel signaling cascade involving CnAß-FK, a constitutively active variant of CnA, that controls the growth of follicular keratinocytes through the regulation of expression of cell-cycle regulatory genes such as cyclin G2 and p21^waf/cip1^.

## Supporting Information

Figure S1 **Full-length nucleotide sequence of human CnAß-FK cDNA.** (A) The underline indicates the ATG initiation codon and the double underline indicates the stop codon. Black, cDNA derived from exons; blue, cDNA derived from introns; red, poly A tail. (B) Comparison of 3′ sequence derived from exon 11 and 12 in CnAß variants. In CnAß-FK cDNA, two sequential stop codons have been inserted at the 3′ end of the cDNA derived from exon 11 (arrow).
(TIF)

Figure S2 **Comparison of the alignment of amino acid sequences among each variant of CnAß.** The thin bar indicates the calmodulin-binding site and the thick bar indicates the autoinhibitory domain (AID). Dashes indicate missing amino acids. CnAß-FK and variant 2 have no autoinhibitory domain.
(TIF)

Figure S3 **Localization of NFATc2 in PHK cells.** Endogenous NFATc2 was observed in both cytoplasm and nucleus of the cells. Scale Bar, 50 µm.
(TIF)

Figure S4 **Expression of CnAß in male rat skin at postnatal day 28.** (A) Endogenous CnAß in hair follicle at a late phase of the anagen stage. (B and C) Higher magnification views of the boxed regions in A. B, epidermal region; C, hair follicle region. CnAß was observed in both epidermal and follicular keratinocytes. Scale bar, 100 µm.
(TIF)

Figure S5 **Distribution of CnAß-FK mRNA and NFATc2 in rat skin at postnatal days 24 and 25 (early phase of the anagen stage).** (A–D) Distribution of CnAß-FK mRNA. (A and B), postnatal day 24 (P24); (C and D), postnatal day 25 (P25). (B and D) Sections were counter-stained with hematoxylin (blue). (E and F) Immunohistochemical analysis of NFATc2 in hair follicles at P24 and P25. In HG cells, NFATc2 was present in the cytoplasm. DP, dermal papillae; HG, hair germ; SG, sebaceous gland. Scale bars, 50 µm.
(TIF)

Figure S6 **Distribution of CnAß-FK mRNA in human skin.** CnAß-FK is also expressed in human hair follicle.
(TIF)

Figure S7 **Effect of CnAß-FK overexpression on cyclin G2 expression in Hela cells.** HeLa cells were transfected with the CnAß-FK/pcDNA3.1-V5-His vector. The cells were harvested 6, 12 and 24 h after the transfection and the amount of cyclin G2 at each time point was measured by Western blotting. V5 is an epitope tag fused with CnAß-FK.
(TIF)

Figure S8 **Cyclosporin A reduces cyclin G2 expression in hair follicles through NFATc2 inhibition.** (A) Time-scale of the hair cycle in male Wister rats. The intensity of gray shading indicates the rate of proliferation of follicular keratinocytes as described in Fig. 3a. (B–E) NFATc2 and cyclin G2 localization in anagen stage hair follicles of a control rat. Arrows indicate nuclear localization of NFATc2. Cyclin G2 was abundant in follicular keratinocytes, similarly to that at P9 (Figure 5E). (F–I) NFATc2 and cyclin G2 in anagen stage hair follicles of a CsA-treated rat. Note that cytoplasmic localization of NFATc2 was observed in many cells (arrowheads in G), resulting in a decrease in cyclin G2 expression in follicular keratinocytes (I). Scale bars, 20 mm.
(TIF)

Table S1 **Primer sequences for cloning of CnAß-FK.** For cloning the full nucleotide sequence of human CnAß-FK cDNA, the PCR amplifications were performed according to the sequential steps **a**, **b**, **c** (described in schema of CnAß-FK mRNA). Details of the experimental procedure are given in the “Materials & Methods”.
(TIF)

Table S2 **Primer sequences for preparation of probes for** ***in situ*** **hybridization.** Anti-sense and sense probes were prepared using a DNA template corresponding to the full sequence of intron11; this sequence was produced using the primers listed in Table S2. Further details of this procedure are given in “Materials and Methods”.
(TIF)
