# Supplementary figures and images for: Expression of a Constitutively Active Calcineurin Encoded by an Intron-Retaining mRNA in Follicular Keratinocytes
Source: PLoS One. 2011 Mar 14;6(3):e17685. doi: 10.1371/journal.pone.0017685 (PMC3056713; doi:10.1371/journal.pone.0017685)

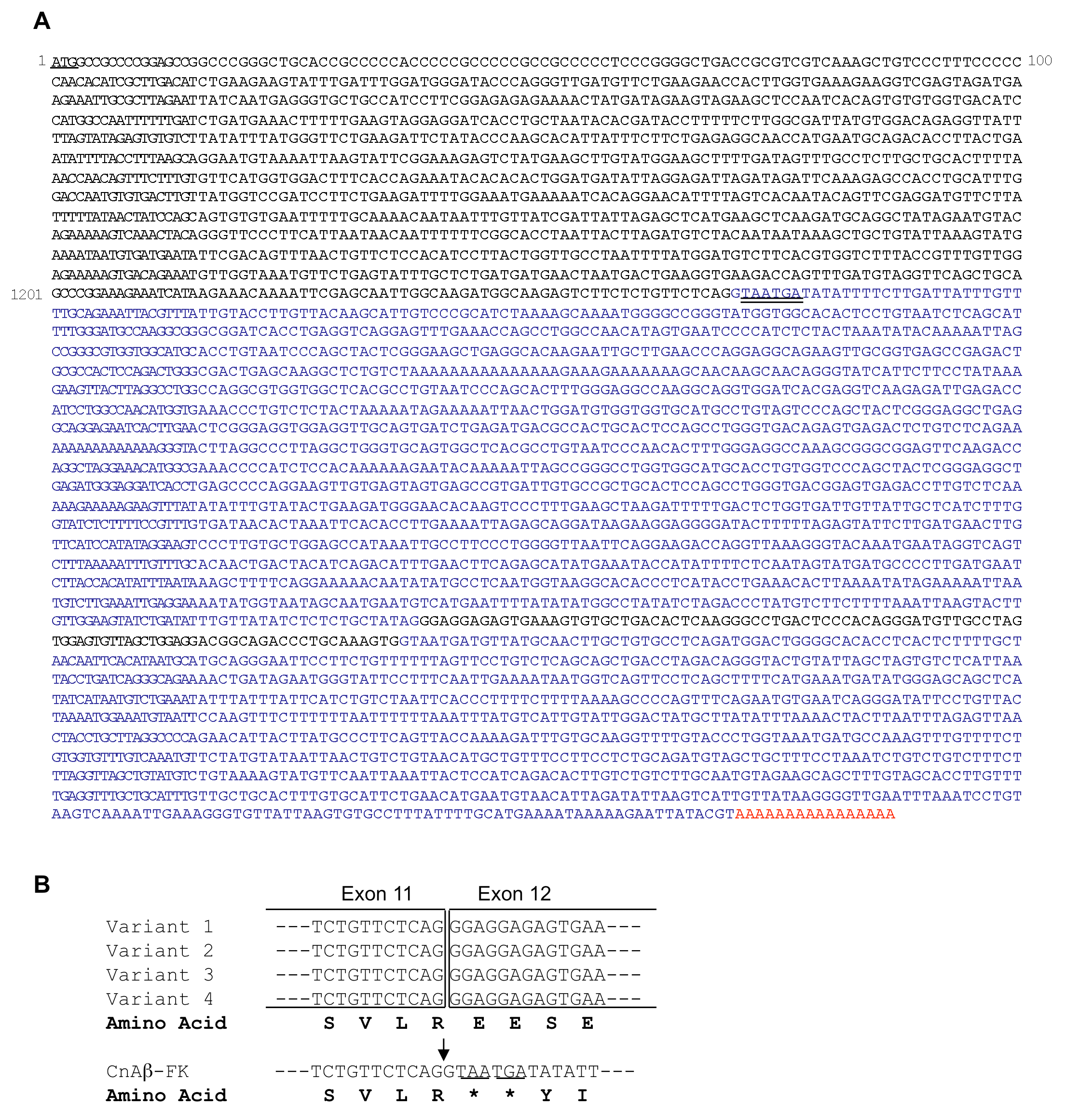

Supplement: Figure S1 — Full-length nucleotide sequence of human CnAß-FK cDNA. (A) The underline indicates the ATG initiation codon and the double underline indicates the stop codon. Black, cDNA derived from exons; blue, cDNA derived from introns; red, poly A tail. (B) Comparison of 3′ sequence derived from exon 11 and 12 in CnAß variants. In CnAß-FK cDNA, two sequential stop codons have been inserted at the 3′ end of the cDNA derived from exon 11 (arrow). (TIF) [file pone.0017685.s001.tif]

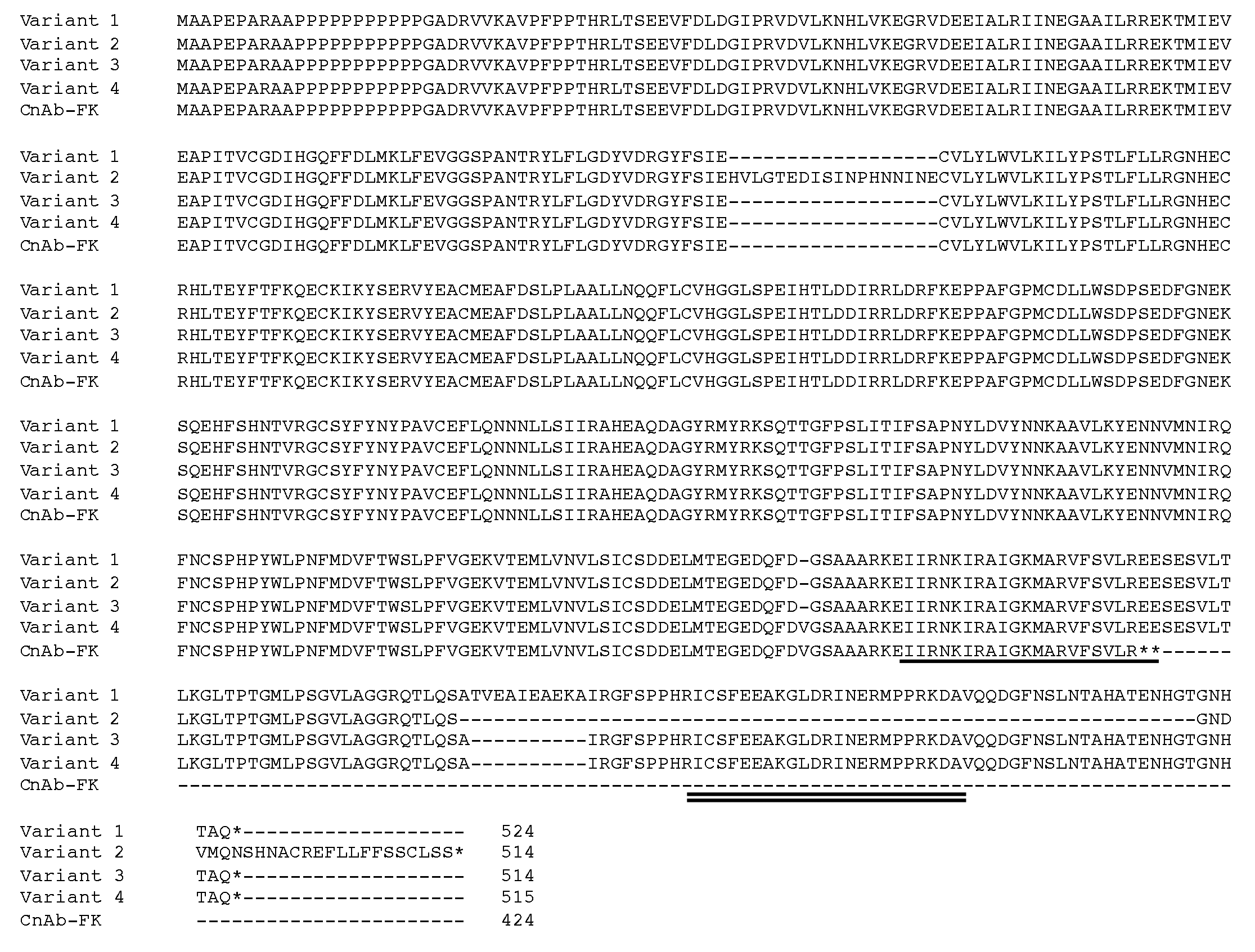

Supplement: Figure S2 — Comparison of the alignment of amino acid sequences among each variant of CnAß. The thin bar indicates the calmodulin-binding site and the thick bar indicates the autoinhibitory domain (AID). Dashes indicate missing amino acids. CnAß-FK and variant 2 have no autoinhibitory domain. (TIF) [file pone.0017685.s002.tif]

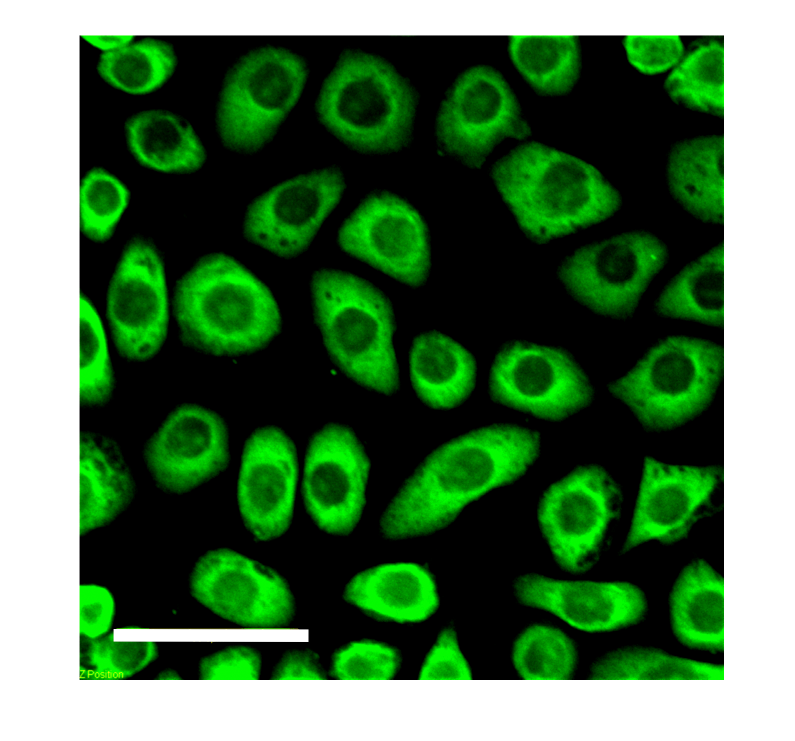

Supplement: Figure S3 — Localization of NFATc2 in PHK cells. Endogenous NFATc2 was observed in both cytoplasm and nucleus of the cells. Scale Bar, 50 µm. (TIF) [file pone.0017685.s003.tif]

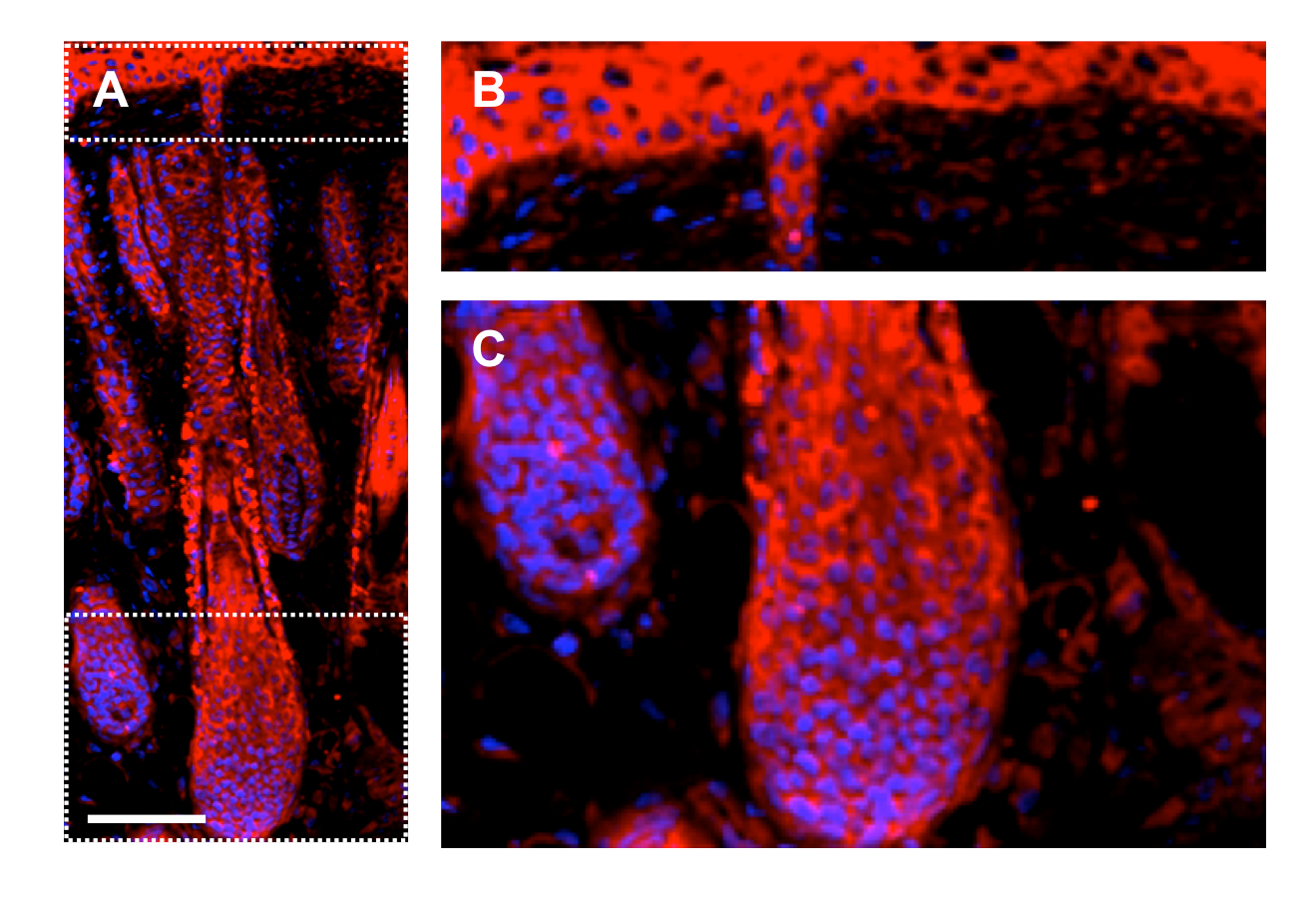

Supplement: Figure S4 — Expression of CnAß in male rat skin at postnatal day 28. (A) Endogenous CnAß in hair follicle at a late phase of the anagen stage. (B and C) Higher magnification views of the boxed regions in A. B, epidermal region; C, hair follicle region. CnAß was observed in both epidermal and follicular keratinocytes. Scale bar, 100 µm. (TIF) [file pone.0017685.s004.tif]

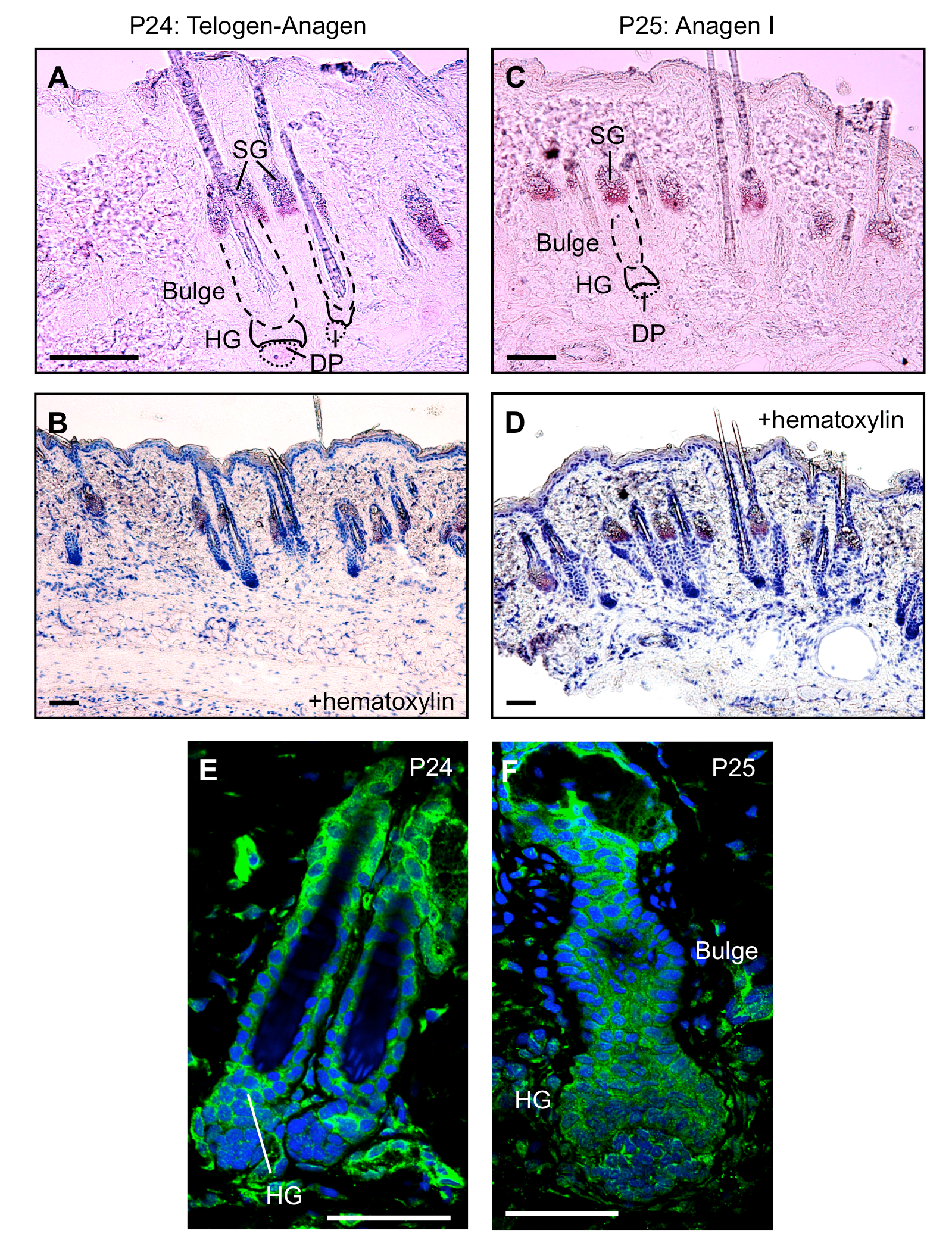

Supplement: Figure S5 — Distribution of CnAß-FK mRNA and NFATc2 in rat skin at postnatal days 24 and 25 (early phase of the anagen stage). (A–D) Distribution of CnAß-FK mRNA. (A and B), postnatal day 24 (P24); (C and D), postnatal day 25 (P25). (B and D) Sections were counter-stained with hematoxylin (blue). (E and F) Immunohistochemical analysis of NFATc2 in hair follicles at P24 and P25. In HG cells, NFATc2 was present in the cytoplasm. DP, dermal papillae; HG, hair germ; SG, sebaceous gland. Scale bars, 50 µm. (TIF) [file pone.0017685.s005.tif]

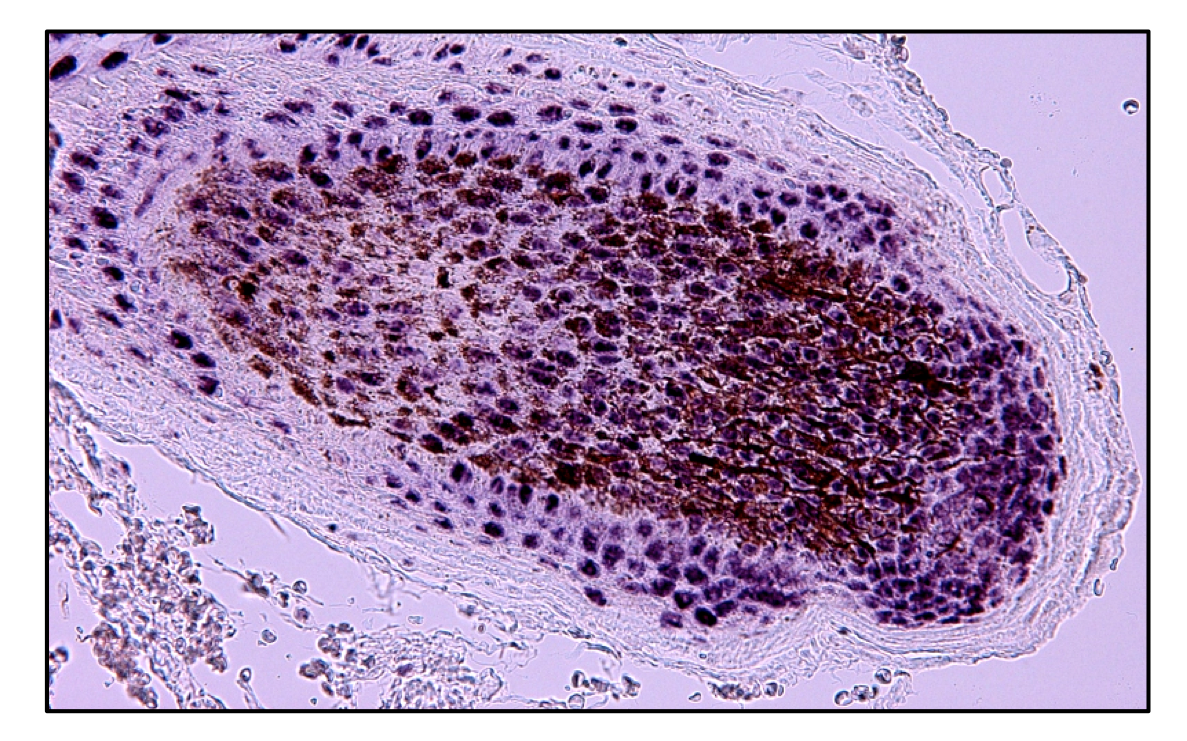

Supplement: Figure S6 — Distribution of CnAß-FK mRNA in human skin. CnAß-FK is also expressed in human hair follicle. (TIF) [file pone.0017685.s006.tif]

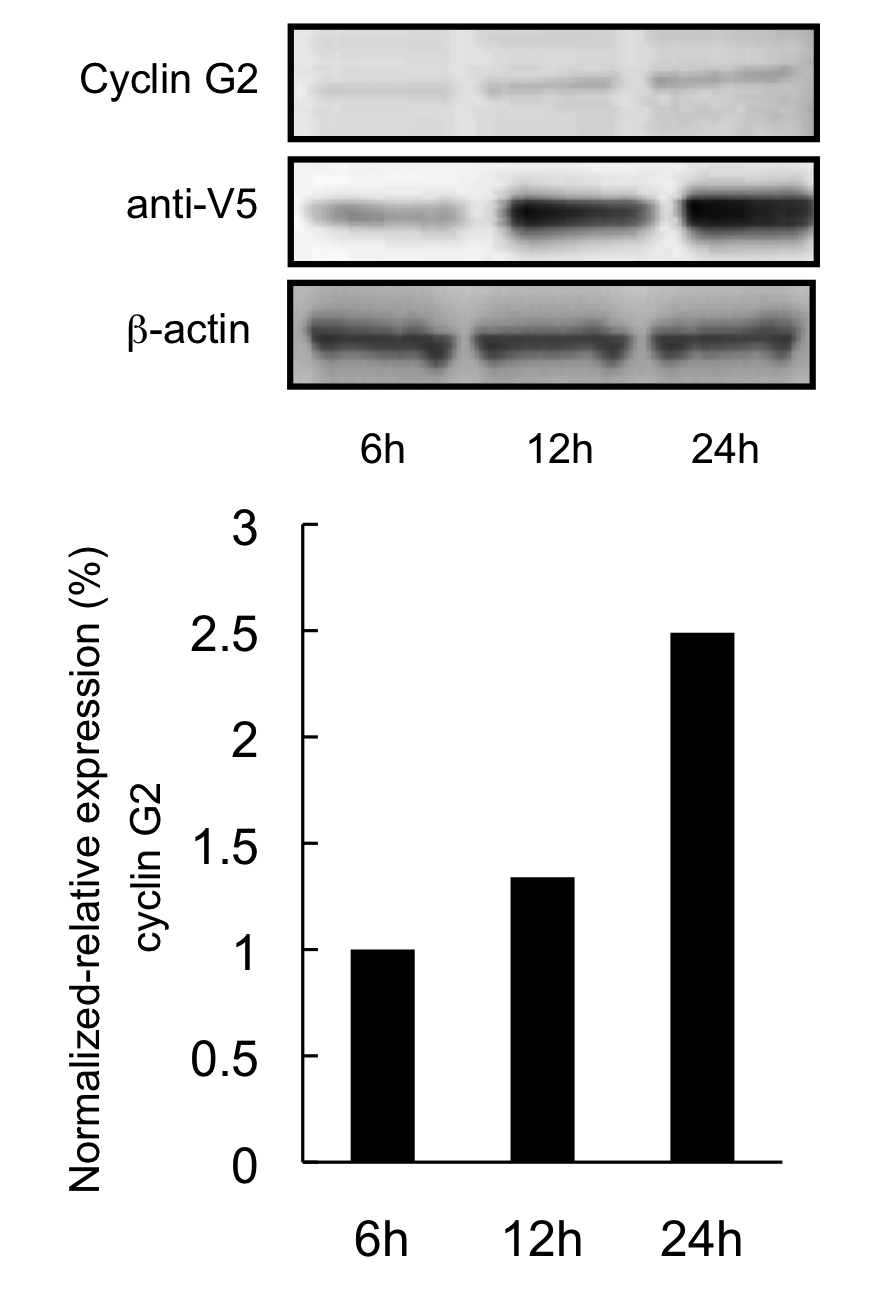

Supplement: Figure S7 — Effect of CnAß-FK overexpression on cyclin G2 expression in Hela cells. HeLa cells were transfected with the CnAß-FK/pcDNA3.1-V5-His vector. The cells were harvested 6, 12 and 24 h after the transfection and the amount of cyclin G2 at each time point was measured by Western blotting. V5 is an epitope tag fused with CnAß-FK. (TIF) [file pone.0017685.s007.tif]

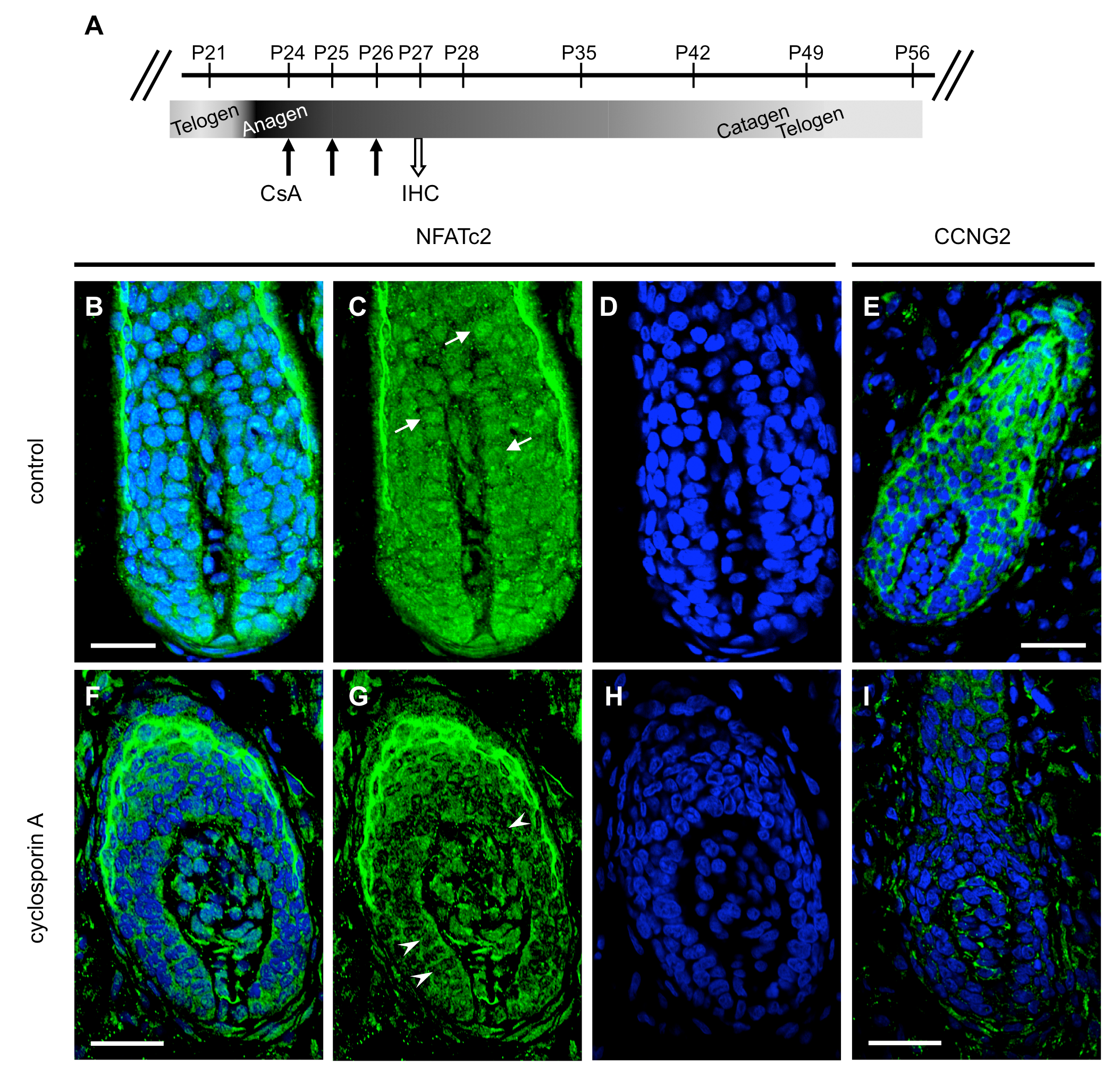

Supplement: Figure S8 — Cyclosporin A reduces cyclin G2 expression in hair follicles through NFATc2 inhibition. (A) Time-scale of the hair cycle in male Wister rats. The intensity of gray shading indicates the rate of proliferation of follicular keratinocytes as described in Fig. 3a. (B–E) NFATc2 and cyclin G2 localization in anagen stage hair follicles of a control rat. Arrows indicate nuclear localization of NFATc2. Cyclin G2 was abundant in follicular keratinocytes, similarly to that at P9 (Figure 5E). (F–I) NFATc2 and cyclin G2 in anagen stage hair follicles of a CsA-treated rat. Note that cytoplasmic localization of NFATc2 was observed in many cells (arrowheads in G), resulting in a decrease in cyclin G2 expression in follicular keratinocytes (I). Scale bars, 20 mm. (TIF) [file pone.0017685.s008.tif]

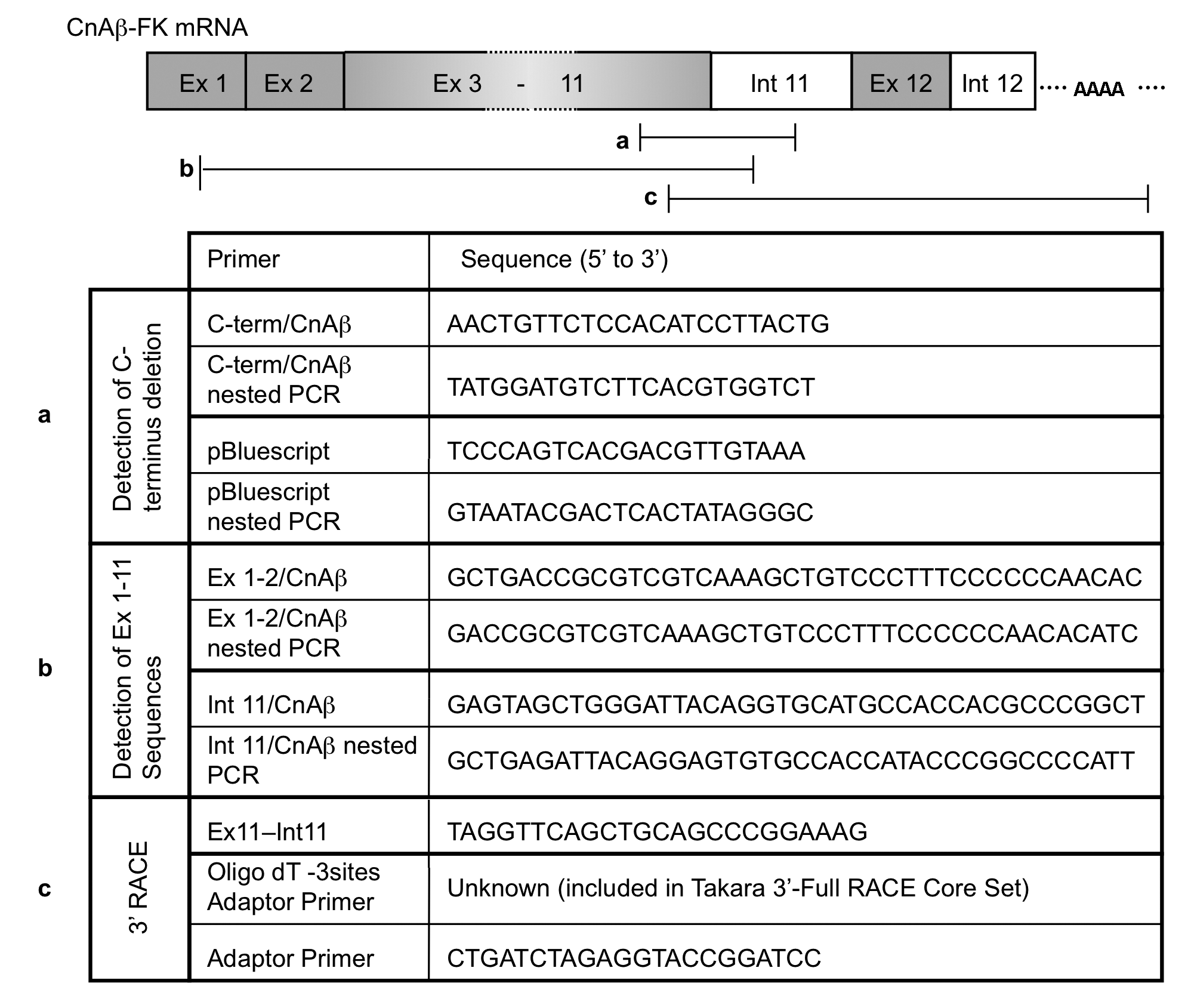

Supplement: Table S1 — Primer sequences for cloning of CnAß-FK. For cloning the full nucleotide sequence of human CnAß-FK cDNA, the PCR amplifications were performed according to the sequential steps a, b, c (described in schema of CnAß-FK mRNA). Details of the experimental procedure are given in the “Materials & Methods”. (TIF) [file pone.0017685.s009.tif]

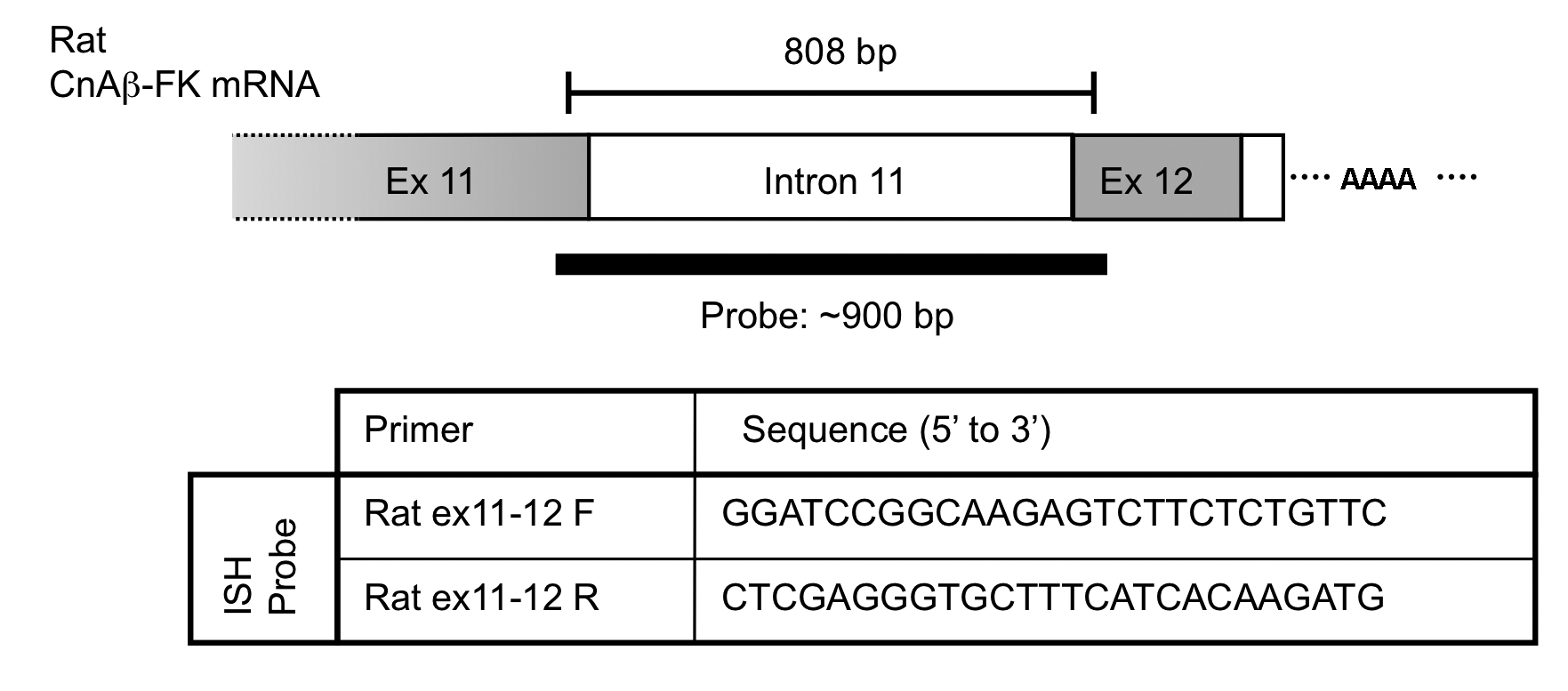

Supplement: Table S2 — Primer sequences for preparation of probes for in situ hybridization. Anti-sense and sense probes were prepared using a DNA template corresponding to the full sequence of intron11; this sequence was produced using the primers listed in Table S2. Further details of this procedure are given in “Materials and Methods”. (TIF) [file pone.0017685.s010.tif]
